# Mysticism

**Published:** 1882-12

**Authors:** J. A. Robinson


### Mysticism.

BY J. A. ROBINSON, D.D.S.

" Draw if thou cans't the mystic line,
Severing rightly his from thine,
Which is human which divine."

—*Emerson.*

On page 548 in *Ohio State Journal* Dr. Watt seems disturbed because we said Dr. Atkinson's paper might be better understood in the future, as Swedenborg in his day was not understood ; but his writings had modified and beautified all the old theologies. Now the question is not whether Atkinson was understood by Prof. Watt or whether Dr. R. is a mystic. It is this, was Dr. Atkinson's paper on nomenclature or philology a mess of nonsense, or was it timely and would it ever become of any use to the profession or the world. Prof. Watt thinks it may be in the end as bad as to return to the " Choctaw gibberish." He also quoted the " Golden Rule " to prove the Disciples of Jesus must have understood Him while on earth. Brother Watt is too familiar with the scriptures not to know that Jesus Himself told His Disciples they did not understand Him. The New Testament is full of His statements that His immediate followers, while they may have understood *His words*, they did not understand what He said, when He said, " the *words* I speak unto you they are spirit, they are life." Neither did they understand the import of the sayings He daily uttered in their presence. " They understood none of these things, neither knew the things that were spoken." " They understood not the sayings He spake unto them." And again He asked them if He had been so long with them, and they could not understand. Paul also tells us, " Behold I show you a mystery." Language is the fruit of the tree of the ages; and the English language is to the American people simple and plain, but the *technical phrases* as belonging to the arts and professions while they are readily understood by scholars, are Greek to the masses and costly and difficult, and an obstruction in this age of railroad and telegraph education of the students and learners. What we said of Swedenborg is true, and while the vocabulary he uses is obscure and difficult, there are no words that express such deep meaning when once understood; *but it costs too much.* His theory of *correspondence* and *degrees* and of *forms*, not only give a new meaning and significance to what he tells us but opens a new field of thought that changes and elevates character and modifies the lives of every person who embraces it, as much as the dead languages give finish and scholarship to the individual mind.

The mystical is an outgrowth of the emotional nature in man; and the emotional nature is as much an entity, a something, as the intellectual or the physical. Words are used to express this emotional nature and acts are the manifestations of the same; because all acts have their beginnings in the mind and the heart. Atkinson has been the great terminologist of the dental profession for the past twenty-five years, and is very often obscure and misunderstood. But we do not believe with Buckle that everything we cannot understand is superstition. The great Daniel Webster in a speech in the United State Senate defeated an appropriation of thirty thousand dollars to help Mr. Morse perfect his telegraphic system, and pronounced it a failure because he could not at that time insulate it and carry it beyond a given point.

There are fifteen hundred new words in the new Webster Unabridged edition and many of them as remote from the old English vocabulary as was the words that Atkinson was trying to introduce As to mysticism, if there is any *real* truth in the world outside of actual material fact, it has its foundation in mysticism. It is the inner life or the soul of man, and mind and speech are reflections of the infinite. The person who sees nothing but so many cords of wood in a tree, or nothing but vapory mist in the clouds, gets very little of nature. To get the whole of nature we must look through and beyond nature. This eternal analysis that goes no further than *analysis* is the ruin of the great heart of man. Everything beyond the bare *material fact* in the world is mystical, and what is not?

In the natural order of progress in man the highest is the mystic, for it is spiritual. When we have made all progress possible in the creeds we outgrow them and become mystical. The characteristic of the present age is analysis; and it tends to skepticism. If the mind can catch an idea of the infinite at all it must be in the mystical. It is only the mystical that can give society of to-day some ideal standard that will save the race. If I have deserved in the least degree the name of a thinker that Dr. Watt has given me, I can assure my brother it has been that in every article I have written, I have always, to the extent of my ability, thrown into what I have said the ideas of Swedenborg and the New Testament.

There lived away off among the Scandinavian hills more than a hundred years ago, a mystic philosopher who, under the patronage of Charles the XII., explored all the mineral, the vegetable and the animal kingdoms of that mountainous land, and brought to the light of science more wonderful discoveries than any person who had gone before him. He did not, like Mr. Darwin, solve the law of evolution, but when he saw the monad and the atom, the amœba and the protoplasm, and the beginning of the natural life that could not be understood by the senses; his mystical soul went out beyond, as it did when he beheld the mountain cliffs of his native land covered with perpetual snow that reached to the clouds, and he began singing a mystical song that is singing in every human heart that begins and ends with this hallowed thought, "There is something beyond and above us." He did not see the connecting link between the fish and the reptile, or between the reptile and the bird, or between the bird and the quadruped, and so up to man, but he saw all these things moving in rapid procession before him and he saw also that the life in them, like the song of his heart, was "there is something above and beyond us." He saw, also, the natural, the spiritual and celestial degrees, and realized the sayings of that other mystic who lived more than eighteen hundred years ago, when he said, " It is not I but my father that dwelleth in me; and he that hath seen me hath seen the father also." And so he sung this song, " There is something above and beyond us that stimulates us to all the divine possibilities of the human heart." This song was in the heart of Columbus when he set sail for the New World, and also in the hearts of the signers of the Declaration of Independence. This song has inspired the pen of Longfellow and Tennyson and Whittier; and also of Fredrick, Bremer and Ole Bull and Jennie Lind. It gave Stevenson the steam engine and Fulton the steamboat, and Morse the telegraph, and Edison the telephone, and the thousand inventors of appliances in dentistry to relieve the world of suffering and harmonize humanity and assist the fittest to survive in the struggle of the human race. It was this same song of " There is something above and beyond us" in the heart of Beethoven that led him to disobey the rules of musical grammar as laid down by Handel and Hayden. It was this same song " There is something above and beyond us" that sung in the heart of Prof. Watt as he toiled over his nightly lamp to give us the chemical analysis of the secretions of the mouth. All positive assurance and dogmatical assertions become silent shadows before this mystical song and we bend our heads in humility before this spiritual phenomena that is always whispering to us words of peace and hope, and saying to us " there is something above and beyond us greater than we." The mystic sees the facts of life coming to him and returning, and knows that they are not him, but something that is revealed to him from the Infinite Father that dwelleth in him. This song has given us our free institutions, our schools and our churches, and sings to us the grand song of Immortality in the words of the great Scandinavian mystic, "There is something above and beyond us."

---

THE art of prevention in disease is the best lesson for the teacher and the student. As the proverb says, " An ounce of prevention is better than a pound of cure." Even the brute creation, as in the marshes near Rome, have, through experiment, symptoms of malaria produced by infection of the soil and air.

